# Supplementary material for: Knowledge, attitude and reported practice regarding donning and doffing of personal protective equipment among frontline healthcare workers against COVID-19 in Nepal: A cross-sectional study
Source: PLOS Glob Public Health. 2021 Nov 12;1(11):e0000066. doi: 10.1371/journal.pgph.0000066 (PMC10021608; doi:10.1371/journal.pgph.0000066)
Supplement: S1 File — (DOCX) [file pgph.0000066.s001.docx]

# Questionnaire

# Demographic details:

1. Age in years

2. Gender: M/F/Other

3. Marital Status: Married/Unmarried/Divorced/Separated

4. Profession: 1). Medical Officer 2). Consultant 3). Staff Nurse 4). BSC Nursing/BN 5). MSC Nursing/MN 6). Others (specify)

5. Work Experience (in Year/Month)

6. Place of Work: 1). Fever Clinic 2). ER 3). COVID-19 ICU/HDU 4). COVID-19 Ward 5). Other (specify)

7. Location: 1). Province 1 2). Province 2 3). Province 3 4). Province 4 5). Province 5 6). Province 6 7). Province 7

8. Does your healthcare institution provide complete set of standard PPE while managing COVID-19 patients? 1). Yes 0). No

9. Does your healthcare institution have a designated space for methodical way of donning and doffing? 1). Yes 0). No

10. Vaccination status against COVID-19: 1). Both dose taken 2). Single dose taken 3). Unvaccinated

11. Place of residence: 1). Own home 2). Rented home 3). Quarter 4). Hostel 5). Others (specify)

12. Are you currently staying with other family members? 1). Yes 0). No

If yes, are there elderly (≥60 years) or pregnant or children (<5 years) in your family?

1). Yes 0). No

# Knowledge

# 1. Have you heard about donning and doffing of PPE for frontline health-care workers?

# 1) Yes 0) No

# 2. Have you received any formal “training or demonstration” regarding donning and doffing of PPE?

# 1) Yes 0) No

# 3. Do you know the complete procedure of donning and doffing of PPE in a health-care facility?

# 1) Yes 0) No

# 4. Arrange the following components based on their sequence of appearance during donning of PPE. 1). Mask or respirator 2). Gowns 3). Gloves 4). Goggles or face shield

# 5. Arrange the following components based on their sequence of removal during doffing of PPE. 1). Mask or respirator 2). Gowns 3). Gloves 4). Goggles or face shield

# 6. Which of the following components of PPE are necessary to wear while drawing venous blood from a COVID-19 patient?

# 1) Gloves only 2) Gloves and mask 3) Gloves, mask and face shield/goggles 4) Gloves, mask, face shield/goggles, and gown

# 7. Which of the following components of PPE are necessary to wear while suctioning oral secretions from a COVID-19 patient?

# 1) Gloves only 2) Gloves and mask 3) Gloves, mask and face shield/goggles 4) Gloves, mask, face shield/goggles, and gown

# 8. Can personal eyeglasses be used as barrier protection for eyes instead of goggles as a form of PPE while managing COVID-19 patients?

# 1) Yes 0) No

# 9. Is it safe to adjust your goggles yourself by your gloved hands after donning of PPE while managing a COVID-19 patient?

# 1) Yes 0) No

# 10. Risk of virus dispersion is highest during?

# 1) Donning of PPE

# 2) Doffing of PPE

# 3) It is same in both the procedures

# Attitude:

# 1. Donning and doffing of PPE is a critical process that must be taken seriously by healthcare professionals.

# 1) Strongly disagree 2) Disagree 3) Neutral 4) Agree 5) Strongly Agree

# 2. Standard method of donning and doffing of PPE can be modified based on convenience.

# 1) Strongly disagree 2) Disagree 3) Neutral 4) Agree 5) Strongly Agree

# 3. Healthcare workers are completely protected from COVID-19 transmission if they use standard PPE even if they do not follow the proper method of donning and doffing of PPE.

# 1) Strongly disagree 2) Disagree 3) Neutral 4) Agree 5) Strongly Agree

# 4. It is reasonable to engage in care of patient with COVID-19 before donning PPE to avoid the inconvenience after PPE use.

# 1) Strongly disagree 2) Disagree 3) Neutral 4) Agree 5) Strongly Agree

# 5. Donning and doffing of PPE is important only while managing patients with COVID-19 and can be ignored while caring for patients with other infectious diseases.

# 1) Strongly disagree 2) Disagree 3) Neutral 4) Agree 5) Strongly Agree

# 6. All healthcare workers should use complete set of standard PPE in all situations no matter the type of anticipated infectious exposure.

# 1) Strongly disagree 2) Disagree 3) Neutral 4) Agree 5) Strongly Agree

# 7. I tend to compromise on standard donning and doffing practice when my colleagues/other healthcare workers do not follow proper way of donning and doffing of PPE.

# 1) Strongly disagree 2) Disagree 3) Neutral 4) Agree 5) Strongly Agree

# 8. Standard practice of donning and doffing of PPE would wear off of the pandemic continues for a long period of time.

# 1) Strongly disagree 2) Disagree 3) Neutral 4) Agree 5) Strongly Agree

# Practice

# 1. Do you always follow standard donning and doffing of PPE practices while taking care of suspected COVID-19 patients?

# 1) Yes 0) No

# 2. Do you do donning/doffing in all patients undergoing any surgery or airway related procedures irrespective of their COVID-19 status?

# 1) Yes 0) No

For questions 3-5: Which of the following instructions do you follow before donning PPE kits?

3) Get enough water intake to remain hydrated.

1) Yes 0) No

4) I always get all my jewelry/mobile or other personal belongings removed.

1) Yes 0) No

# 5) I always sanitize my hands, before touching any PPE component.

1) Yes 0) No

# For questions 6-11: Which of the following things do you do during the donning procedure of PPE kits?

# 6) I always perform donning procedures before entering the patient’s room.

1) Yes 0) No

# 7) I always visually check the integrity of the components of PPE kits before donning procedure.

1) Yes 0) No

# 8) I always perform hand hygiene during donning PPE.

1) Yes 0) No

# 9) I always put the gown first before putting on the first pair of gloves.

1) Yes 0) No

# 10) I always use a respirator or N95 mask followed by eye goggles/face shield.

1) Yes 0) No

# 11) I move out of the patient care area after donning PPE.

1) Yes 0) No

# For questions 12-17: Which of the following things do you do “during doffing” procedure of PPE kits?

# 12) I always use a specified allocated area in my healthcare facility for doffing of PPE.

1). Yes 0). No

# 13) I always remove gloves first during the doffing procedure using glove-in-glove technique without sanitizing the gloves.

1). Yes 0). No

14) I remove the gown after removing the inner pair of gloves.

1). Yes 0). No

15) I turn the gown inside-out during removal to get the infected side packed inside of the gown.

1). Yes 0). No

16) I move out from the doffing area after removal of gloves and N95 mask.

1). Yes 0). No

17) I sanitize my hands/gloves before and after each step of doffing procedure.

1). Yes 0). No
