# Supplementary material for: Knowledge, attitude and reported practice regarding donning and doffing of personal protective equipment among frontline healthcare workers against COVID-19 in Nepal: A cross-sectional study
Source: PLOS Glob Public Health. 2021 Nov 12;1(11):e0000066. doi: 10.1371/journal.pgph.0000066 (PMC10021608; doi:10.1371/journal.pgph.0000066)
Supplement: Attachment — Submitted filename: Response to Reviewers.docx [file pgph.0000066.s002.docx]

2nd October, 2021

The Editorial team,

PLOS Global Public Health.

Dear Editor and Reviewers,

We would like to thank PLOS Global Public Health team of editors and reviewers for providing valuable comments to improve the article on "Knowledge, attitude and reported practice regarding donning and doffing of Personal Protective Equipment among frontline healthcare workers against COVID-19 in Nepal: A cross-sectional study." We have tried to respond to each of the points raised by the editor and reviewers in subsequent section of this letter. Besides the tracked changes visible in the manuscript, we have tried to include line numbers as per manuscript without revised changes to spot the edits easily.

**Editor Comments:**

1. Additional details about the questionnaire is added in the section “Study tool” (142,143 and 151-156). And the questionnaire developed as a part of this study is included in supplementary file “S2 File Questionnaire”.

We have also reviewed the reference list for its completeness and correctness as well as to ensure it is according to the journal preferences. We have not cited any retracted papers in the article and the reference list is the same as the one in the original submitted manuscript except the minor corrections which are highlighted in tracked changes. Also the reference in position “16” is moved to “17” and that at “17” is moved to position “16” in the process of refining the description of “Study tool”.

Additional Editor Comments:

I have reviewed the language of the paper to make it grammatically correct and present the information in a clear and concise manner within journal recommendation. Reference section of the manuscript is also corrected accordingly without adding or deleting the existing list of references.

**Reviewers’ Comments:**

**Reviewer#1:**

-Call for study details, especially the ethical considerations is elaborated further in more details is included in “Ethics” section in lines 193-199 in revised manuscript.

-Regarding registry system used for sampling, we used the list of healthcare workers prepared by eight investigators from their social media contacts. All of the healthcare workers belonged to different parts of the country, and had a diverse group of healthcare workers as their contacts which was used for sampling.

-Other factors associated with the scores are elaborated further in the discussion section (365-367 and 372-375).

-Main statistical test (Chi square test) is noted at the end of respective tables.

-Regarding questionnaire, we have elaborated further about the questionnaire details in “Study tool” (142,143 and 151-156) and included a copy of the questionnaire as “S2 File Questionnaire”

**Reviewer#2:**

Abstract

1. Background is shortened

2. Line 55 and 56 are deleted from the conclusion.

Methods:

1. “Study size” is changed to “Sample size” in line 110.

2. We increased the sample size to adjust non-response using the formula:

$$Minimum required sample size=\frac{Calculated sample size}{1-anticipated non response rate}$$

$$Minimum required sample size=\frac{182}{1-0.25} i.e.243$$

This ensured that even if there is 25% non-response in final estimated sample of 243, we get our required minimum sample i.e.182.

However, simply increasing the sample size by 25% of 182 to get a final sample of 228, won’t be able to adjust for 25% non-response in final sample of 228.

3. I have revised the “Sampling technique” portion of the manuscript to address the ambiguous statements (120-129). Eight investigators were involved in compiling the list of frontline healthcare workers and a total of 476 healthcare workers were listed in total by eight investigators. Number of healthcare workers compiled in each of the list ranged from a minimum of 54 to a maximum of 68.

Regarding stratified sampling, we agree that stratified sampling would be better but our study population could not be stratified.

4. Regarding the discrepancy between the minimum sample size and the number of randomly included participants, we conducted an online survey to collect the data amidst the raging second wave of COVID-19 in Nepal where healthcare workers were oftentimes overwhelmed. Anticipating higher chances of non-response, we randomly selected 35 participants from each of the list of eight investigators generating a total number of 280 participants which is higher than the anticipated sample size of 243.

Table:

1. We have removed the workplace province from Table 3 and 4. However, in order to demonstrate the inclusiveness of participants from all of the seven provinces, we want to keep the “workplace province” in “Table 1: Distribution of socio-demographic and workplace characteristics of the participants (n = 205)”.

Discussion:

1. We realized the lack of clarity and probable ambiguity in the explanation given for discrepancy in findings in lines 291-294 in original manuscript and therefore have eliminated the statement.

Conclusion:

1. Conclusion has been revised to address the study findings (389-394). Discussion has been revised to address the recommendations mentioned in the conclusion (337-339).

We hope we addressed the comments. We always look forward to any additional recommendations to improve the article.

Sincerely,

Dr. Sagar Pandey

Corresponding author.
